# Measurement of provider fidelity to immunization guidelines: a mixed-methods study on the feasibility of documenting patient refusals of the human papillomavirus vaccine

**DOI:** 10.1186/s12911-022-02083-2

**Published:** 2022-12-22

**Authors:** Rachel S. Chang, Jaimie Z. Shing, Jennifer C. Erves, Liping Du, Tatsuki Koyama, Stephen Deppen, Alyssa B. Rentuza, Caree McAfee, Christine Stroebel, Janet Cates, Lora Harnack, David Andrews, Robert Bramblett, Pamela C. Hull

**Affiliations:** 1 School of Medicine, Vanderbilt University, Nashville, TN USA; 2 Division of Epidemiology, Department of Medicine, Vanderbilt University Medical Center, Nashville, TN USA; 3 Department of Internal Medicine, Meharry Medical College, Nashville, TN USA; 4 Department of Biostatistics, Vanderbilt University Medical Center, Nashville, TN USA; 5 Markey Cancer Center, University of Kentucky, 2365 Harrodsburg Rd, Suite A230, Lexington, KY 40504-3381 USA; 6 Cumberland Pediatric Foundation, Nashville, TN USA; 7 Department of Behavioral Science, University of Kentucky, Lexington, KY USA; 8 Sharecare, Atlanta, GA USA

**Keywords:** Measurement, Fidelity, Clinical guidelines, Assessment and feedback, Vaccine refusal

## Abstract

**Background:**

Assessment and feedback is a common implementation strategy to improve healthcare provider fidelity to clinical guidelines. For immunization guidelines, fidelity is often measured with doses administered during eligible visits. Adding a patient refusal measure captures provider fidelity more completely (i.e., all instances of a provider recommending a vaccine, resulting in vaccination or refusal) and enables providers to track patient vaccine hesitancy patterns. However, many electronic health record (EHR) systems have no structured field to document multiple instances of refusals for specific vaccines, and existing billing codes for refusal are not vaccine specific. This study assessed the feasibility of a novel method for refusal documentation used in a study focused on human papillomavirus (HPV) vaccine.

**Methods:**

An observational, descriptive-comparative, mixed-methods study design was used to conduct secondary data analysis from an implementation-effectiveness trial. The parent trial compared coach-based versus web-based practice facilitation, including assessment and feedback, to increase HPV vaccination in 21 community-based private pediatric practices. Providers were instructed to document initial HPV vaccine refusals in the EHR's immunization forms and subsequent refusals using dummy procedure codes, for use in assessment and feedback reports. This analysis examined adoption and maintenance of the refusal documentation method during eligible well visits, identified barriers and facilitators to documentation and described demographic patterns in patient refusals.

**Results:**

Seven practices adopted the refusal documentation method. Among adopter practices, documented refusals started at 2.4% of eligible well visits at baseline, increased to 14.2% at the start of implementation, peaked at 24.0%, then declined to 18.8%. Barriers to refusal documentation included low prioritization, workflow integration and complication of the billing process. Facilitators included high motivation, documentation instructions and coach support. Among adopter practices, odds of refusing HPV vaccine were 25% higher for patients aged 15–17 years versus 11–12 years, and 18% lower for males versus females.

**Conclusions:**

We demonstrated the value of patient refusal documentation for measuring HPV vaccination guideline fidelity and ways that it can be improved in future research. Creation of vaccine-specific refusal billing codes or EHR adaptations to enable documenting multiple instances of specific vaccine refusals would facilitate consistent refusal documentation.

*Trial Registration* NCT03399396 Registered in ClinicalTrials.gov on 1/16/2018.

## Background

Assessment and feedback, also known as audit and feedback, is an implementation strategy commonly used to improve healthcare provider fidelity to clinical guidelines, which involves collecting and summarizing process and outcome measures over time and providing them to providers and operational staff to monitor and inform their quality improvement (QI) efforts [1, 2]. This strategy is often used in combination with other implementation strategies, such as provider training, provider prompts, and patient education [3, 4]. However, the operationalization of appropriate and useful process and outcome measures relevant to specific guidelines can present methodological challenges for generating assessment and feedback reports with repeated measurement over time to inform and evaluate QI efforts [5].

When studying the implementation of clinical guidelines for immunization, patient population coverage rates, defined as the proportion of all active patients in a practice or clinic within a relevant age range who have received the recommended number of doses of a specific vaccine at a given point in time, can be calculated using structured fields in electronic health records (EHRs) or billing codes and used to measure clinical effectiveness outcomes over time [6, 7]. In addition, visit-level process measures can be calculated using structured fields in EHR systems or billing codes to assess the implementation outcome of provider guideline fidelity during specific time intervals; namely, the rate at which a provider performs appropriate application of the clinical guideline during each patient visit. When visit-level process measures are tracked over time in assessment and feedback reports, they can help providers monitor the impact of their practice changes and plan additional changes during QI efforts.

With respect to human papillomavirus (HPV) vaccination, this is commonly measured as the proportion of patient visits with a dose of HPV vaccine administered, among all patient visits that occurred in a given time period in which the patient was due for a dose [8, 9]. Facilities that report vaccinations to their public health department’s immunization information system (IIS) can use the IIS to generate immunization coverage rates for their patient population as effectiveness outcome measures for specific vaccines [10], but an IIS does not contain data about patient visits in which vaccines were not administered, which would be necessary to calculate a comparable visit-level measure [11, 12]. Some EHR systems offer healthcare facilities the ability to generate immunization coverage rate reports for their patient population that can be used as effectiveness outcome measures [6], but few EHRs offer report templates for visit-level measures or the ability to customize vaccination process measures for visits within specific time periods [5]. Existing International Classification of Diseases, 10^th^ Revision, Clinical Modification (ICD-10-CM) diagnostic codes related to vaccine refusals have limited utility for assessment and feedback reports because they do not indicate which specific vaccine or multiple vaccines were refused in a given visit [13].

Current national guidelines recommend routine HPV vaccination for male and female adolescents aged 11–12 years, with catch up (late) vaccination for older adolescents and young adults through age 26, plus optional vaccination for some patients through age 45 [14]. HPV vaccine uptake remains suboptimal in the US, with HPV vaccine initiation (1 + dose) and up-to-date (all dose) coverage of 75.1% and 58.6%, respectively, among adolescents aged 13–17 years in 2020 [15]. Provider recommendation consistently ranks among the strongest patient-level predictors of HPV vaccine uptake, while parental hesitancy has been identified as a significant barrier to increasing HPV vaccination coverage [16–18]. Some implementation intervention studies have examined visit-level process measures of HPV vaccine administration during visits in which patients were due for the vaccine, or the inverse, the proportion of patient visits in which a dose of the HPV vaccine was due but *not* administered, often referred to as a “missed opportunity” [7, 19–21]. However, the lack of administering an HPV vaccine dose in a visit does not accurately reflect whether providers *missed the opportunity to recommend the HPV vaccine* for a patient because it does not capture visits in which the provider recommended HPV vaccine but the parent/patient declined it. Given the importance of provider recommendation and the problem of HPV vaccine hesitancy, another potentially useful visit-level process measure of provider guideline fidelity would be to determine how often providers *recommend* HPV vaccine during visits in which the patient is due for the vaccine, regardless of whether the vaccine is ultimately *administered or refused* by the patient in that visit.

Some previous studies have recorded or observed provider-patient interactions to determine how often and what communication style providers use to recommend vaccines and whether parents accept or refuse vaccination [22, 23]. However, this labor intensive, qualitative approach is not feasible or sustainable as a data source for ongoing assessment and feedback reports related to provider recommendations and patient refusals. In addition, many physicians write free-text comments about vaccine refusals in the clinical notes section of an individual patient’s visit in the EHR for reference in future visits, but unstructured free-text in clinical notes is time-consuming to review manually for repeated measurements over time and difficult to incorporate into assessment and feedback data reports that summarize all eligible visits or patients in a clinic [21, 24]. While emerging informatics research has begun to explore natural language processing methods to extract information from free-text clinical notes for specific purposes, such as classifying diseases or identifying vaccine-related adverse events [25–27], literature searches did not reveal any research that has validated natural language processing methods to classify vaccine refusals from free-text clinical notes. If the necessary information existed within structured, searchable fields in EHRs or billing codes, they could be leveraged to efficiently generate a visit-level measure of provider recommendation of HPV vaccine for use in assessment and feedback reports; e.g., calculated with a numerator counting the number of visits with HPV vaccine administered *plus* the number of visits with HPV vaccine refused, divided by the denominator of eligible visits in which HPV vaccine was due. However, previous research has not examined the feasibility and usefulness of using structured fields in EHRs or billing codes to measure provider recommendation of HPV vaccine or patient refusals of HPV vaccine, likely due to challenges in the way that vaccine refusals are documented with current billing codes and in the diverse array of commercial EHRs [28].

Given these limitations, new measurement methods are needed to document instances of patient refusals of HPV vaccine so refusals can be incorporated into assessment and feedback reports to track documented instances of providers recommending the vaccine. Visit-level measures on both HPV vaccine administration and refusals would provide valuable information to help providers understand changes in their vaccination rates over time and guide them on whether to focus ongoing adaptations of their QI strategies on gaps in *offering* HPV vaccine to patients versus problems with patient *refusals*. In addition, a patient refusal measure could be used to understand demographic patterns in patient refusals, inform strategies to reduce refusals, and evaluate the effectiveness of implementing such strategies. One in three parents self-reported in a national survey that they had ever delayed or refused HPV vaccination for their adolescent children when recommended by a healthcare provider in the past [29]. However, patterns of patient refusals of HPV vaccine have only been studied through provider or patient surveys [29–32]. Published research to date has not reported on the prevalence of or demographic patterns in patient refusal of HPV vaccine during actual healthcare visits.

Within a larger practice facilitation implementation-effectiveness trial aimed at increasing HPV vaccination in community-based pediatric practices, our team implemented a novel measurement method to enable providers to document HPV vaccine refusals for inclusion in assessment and feedback reports to inform QI projects. The objectives of the current methodological analysis were: (1) to assess the feasibility of implementing this refusal documentation method by (a) identifying correlates of successful adoption of refusal documentation among study practices, (b) examining patterns of adoption and maintenance over time, and (c) identifying barriers and facilitators of adoption; and (2) to demonstrate the value of this refusal documentation method for examining demographic patterns in patient refusals of the HPV vaccine. This is the first published methodological analysis of an approach to document multiple instances of HPV vaccine refusal using structured, searchable fields in a context with multiple existing EHRs. The overall goals were to inform the future refinement of refusal measurement methods for implementation research focused on HPV vaccine and other vaccines, and to inform future adaptations to EHRs or billing codes that can enable sustainable documentation of multiple vaccine refusals in structured fields and support vaccine QI efforts.

## Methods

### Study design

An observational, descriptive-comparative mixed-methods study design was used to conduct secondary analysis of data from a hybrid type-2 effectiveness-implementation trial [33] for the purpose of assessing the feasibility and utility of measuring patient refusals of HPV vaccine during clinic visits for inclusion in assessment and feedback reports to support QI projects. The parent trial used a cluster randomized design to compare two modes of delivering practice facilitation (Coach-Based versus Web-Based) for QI projects to increase HPV vaccination among adolescents aged 11–17 years (ClinicalTrials.gov #NCT03399396). The main trial results are forthcoming.

### Parent study setting and practice facilitation implementation

Cumberland Pediatric Foundation is a non-profit membership organization with over 80 community-based private pediatric practices in the state of Tennessee, USA, and is affiliated with the Monroe Carell Jr. Children’s Hospital at Vanderbilt. Cumberland Pediatric Foundation announced the opportunity to participate in the study to all member practices. Twenty-five practices expressed interest, three were not included in the trial because they did not use an EHR, one decided not to participate, and 21 practices agreed to participate in the trial. Participating practices were randomly assigned to either a Coach-Based (10 practices) or Web-Based (11 practices) practice facilitation arm. The study practices completed two consecutive QI projects lasting 6-months each. Each practice identified an internal “Physician Champion” and an “Operations Champion” based on their role (physician or operational staff) who was willing to volunteer to lead and oversee implementation activities within the practice. The Coach-Based arm consisted of an in-person QI coach directly delivering all practice facilitation components to the Champions, including discussion of assessment and feedback reports. The Coach was hired for this project and was selected based on experience in quality improvement, clinical workflows, and practice facilitation in primary care settings. The Web-Based arm consisted of an interactive website that provided all of the same content that the Coach-Based arm received, guided the Champions to implement the same QI process within their practices, and provided the assessment and feedback reports for the Champions to download and review on their own. Based on the timing of study enrollment, practices began their Implementation Phase either in January, February, or March of 2018. The full details of the practice facilitation implementation will be described in a forthcoming publication with the main trial results. The current analysis focused solely on assessing the methodology for documentation of patient refusals of HPV vaccination used in the assessment and feedback reports.

### Review of existing options for providers to document vaccine refusals

During the study planning phase, the team met with physician and operational leaders in each practice to discuss plans for the study and gather their input on typical clinical workflows and the process for extracting data from the EHR for the assessment and feedback reports, including their current method of documenting HPV vaccine refusals and suggestions on options to capture these refusals. They indicated that typical practice workflows for pediatric well visits consisted of the following steps: The patient checks in at the front desk and is called back to an exam room, a nurse or medical assistant does intake following an EHR visit template and may make a note for the provider if any immunizations are due, then a provider sees the patient. None of the participating practices used standing orders for designated clinical staff to recommend and administer the first dose of vaccines prior to the provider seeing the patient. They indicated that immunizations are one of many standard components of pediatric well visits at certain ages, so providers routinely look at the EHR immunization grid to check the patient’s immunizations history and decide which vaccines to recommend; some practices also enable EHR prompts to alert providers to vaccines that are due. If the parent/patient declines a recommended vaccine, some providers make free-text comments about vaccine refusals in the clinical notes for future reference, and some providers may document refusal of a specific vaccine in the immunization grid if the designated space is empty (see below); and less commonly, some providers may use ICD-10-CM billing codes exist under category Z28 to indicate if any vaccines were not administered (see below). If the patient agrees to the recommended vaccines, the provider documents the dose orders in the EHR, and a nurse or medical assistant administers the injections. Then the patient checks out.

The practices indicated that, among the 10 different EHRs used by the study practices, all the EHRs had an immunization grid with only one date field for each dose of each vaccine. This date field could be used to indicate either the date the dose was administered, the date it was refused by the patient, or the date it was determined to be contraindicated; contraindication is rare for HPV vaccine. A date in this field could be replaced, but multiple refusal dates could not be added for the same vaccine dose in the immunization grid. They pointed out a limitation of these EHRs that they did not include another structured field inside the immunization grid or elsewhere for documenting more than one instance of a patient refusing a dose of a specific vaccine.

Some of the practices mentioned that providers often made note of initial and subsequent refusals in free-text clinical notes, but to our knowledge, no validated natural language processing tools were available at the time to classify HPV vaccine refusals from unstructured clinical notes to incorporate into data reports. A handful of practices mentioned that they used the ICD-10-CM billing codes exist under category Z28 (Immunization not carried out and under-immunization status) to document when one or more vaccines were not given during a specific visit due to patient refusal, contraindication or provider decision to defer vaccination, but they noted the limitation that these codes are not vaccine specific [13]. Thus, it would be impossible to infer accurately from the Z28 codes whether or not multiple vaccines were offered in each visit and whether the HPV vaccine specifically was refused. The practices did not report any other existing methods of documenting specific vaccine refusals using structured fields or codes. Given the limitations of the existing options, the study team determined that a new method would need to be designed to document specifically HPV vaccine refusals during patient visits for the purpose of generating assessment and feedback reports for practices’ QI projects in the study.

### New method used in study to document patient refusals of HPV vaccine

**Fig. 1:**
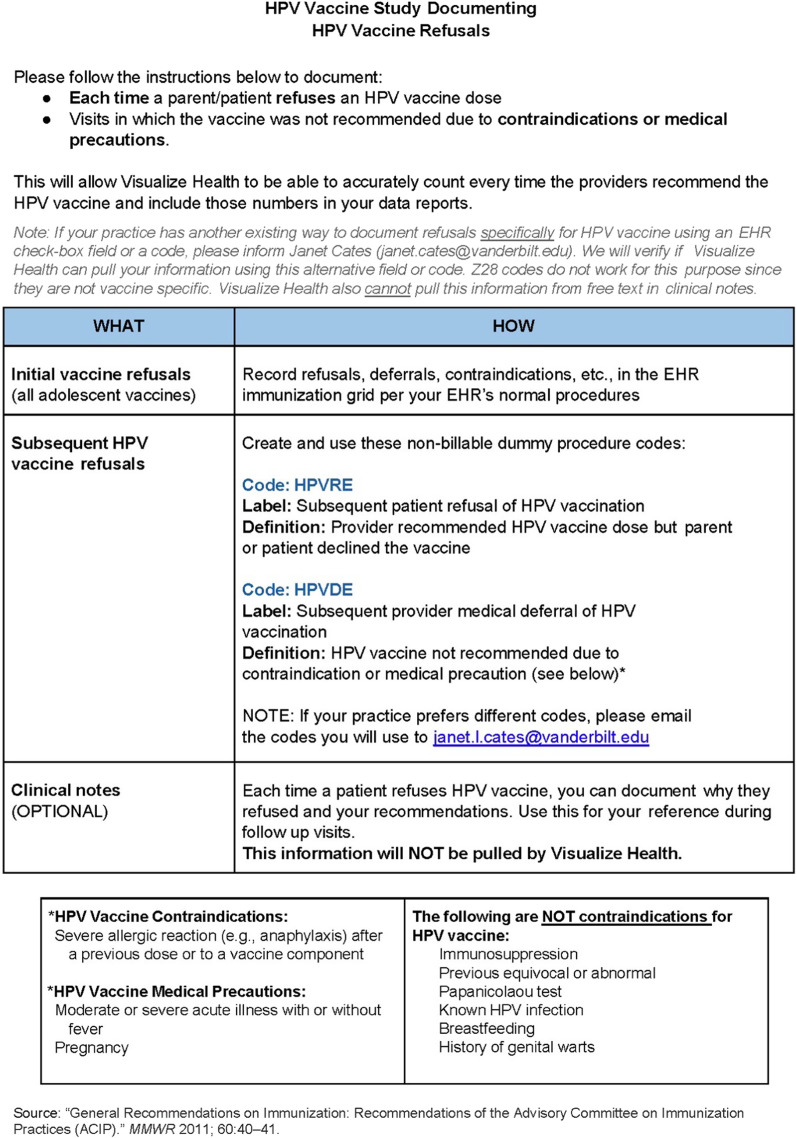
Instructions provided to study practices on how to document HPV vaccine refusals

For the parent study, the study team contracted a third-party vendor (Sharecare, Atlanta, GA, USA) to provide data extraction and visualization support using their software application called Visualize Health, which they installed for each study practice. With input from the participating practices and Sharecare staff, we designed a new method for documenting HPV vaccine refusals within a typical patient visit workflow, and we gave practices the option to customize their own preferred method using structured fields or codes. In consultation with Sharecare staff, the study team created a handout (Fig. 1) with instructions on how providers should document patient refusals of HPV vaccine during the study and made it available to each practice. In the handout, providers were instructed to document each time they offered an HPV vaccine dose and it was refused by the patient or parent, as well as each time a provider deferred recommending HPV vaccine due to a medical contraindication or medical precaution. *Initial* vaccine refusals and deferrals were to be recorded in the EHR’s immunization grid using the EHR’s existing procedure. Practices were told to record *subsequent* HPV vaccine refusals or deferrals using a non-billable “dummy” procedure code (“HPVRE” for refusals; “HPVDE” for medical deferrals) so the data could be extracted from the EHR and billing system using the Visualize Health application. We selected this approach because Sharecare and Cumberland Pediatric Foundation practices had successfully used non-billable dummy procedure codes in a previous QI project on another topic to track a targeted provider action, and dummy codes have been used in other contexts to track information about visits when relevant fields or codes do not exist [34]. In addition, the handout indicated that practices should contact the study team if they already used a different approach to document refusals specifically for HPV vaccine in a structured field, so that Sharecare could customize data extraction and reporting for those participating practices.

In the Coach-Based arm, the QI coach provided this handout and discussed the instructions at the first meeting with Champions at the beginning of the first QI project and then during each subsequent Coach meeting to inquire about their progress with documenting refusals and give feedback, including sharing tips and strategies used by other practices. Champions in Web-Based practices were prompted to download the handout from their web portal in the first module at the beginning of the first QI project and then again as a reminder in each subsequent module. Sharecare offered a technical assistance visit to the Web-Based practices to assist with utilizing the Visualize Health dashboard and documenting refusals, and they shared successful tips used by other practices. All practices were instructed that they could also request technical assistance from Sharecare regarding refusal documentation at any time via the Coach or the web portal.

Over the course of the 12 months, each practice received periodic run chart data reports for their own practice that included general instructions on how to interpret them, and quarterly each practice received an assessment and feedback report that included control charts and written comments with their data interpretation and suggestions. In the Coach-Based arm, the Coach discussed the assessment and feedback reports with the Champions during in-person quarterly meetings, including the observations regarding refusal documentation. In the Web-based arm, the Champions were prompted via email to download and read the quarterly assessment and feedback reports, then discuss them with the providers in their practice. The quarterly assessment and feedback reports included statements encouraging practices to either start or continue documenting refusals, depending on whether or not evidence of refusal documentation was noted in their data, as well as reminders that they could request technical assistance from Sharecare regarding refusal documentation at any time.

### Data sources and measures

*Aggregated Data from Visualize Health.* Aggregated data reports for each practice were generated with the Visualize Health software during a 12-month Baseline Phase and during the 12-month Implementation Phase in which the practices implemented their two 6-month QI projects. For the aggregate measure of *HPV Refusals in Well Visits*, the denominator was the number of well visits in a given month for active patients ages 11–17 who were *due* for an HPV dose prior to the visit; and among this set of well visits, the numerator was the number of well visits with documentation that HPV vaccine was refused (either in the EHR immunization grid or using the “HPVRE” non-billable dummy code). The aggregate numerator and denominator counts for each practice were also broken down by the following patient demographic subgroups (Table 1): age group (11–12 years, 13–14 years, and 15–17 years), gender (if available), and race/ethnicity (if available). Active patients were defined as those with at least one visit to the practice in the previous three years.

**Table 1:** Practice and patient characteristics by adoption of patient refusal documentation status

	Overall n (%)	Adoption of Patient Refusal Documentation^a^		*p*-value
		Adopters n (%)	Non-Adopters n (%)	
*Practice characteristics*				
Total Number of Practices	21 (100.0)	7 (33.3)	14 (66.7)	–
Study arm				0.537
Coach-based	10 (48.0)	4 (57.0)	6 (43.0)	
Web-based	11 (52.0)	3 (43.0)	8 (57.0)	
Urbanicity				0.717
Town/Rural	5 (24.0)	2 (29.0)	3 (21.0)	
Urban City	16 (76.0)	5 (71.0)	11 (79.0)	
Number of Providers^b^, Mean ± SD	8.8 ± 8.7	7.1 ± 5.0	9.6 ± 10.1	0.767
*Patient population characteristics*^b^				
Total Number of Patients	67,467	17,797	49,670	–
Patient age group				
11–12 years	22,280 (33.3)	6407 (36.2)	15,873 (32.2)	** < 0.01**
13–14 years	20,113 (30.1)	5335 (30.2)	14,778 (30.0)	
15–17 years	24,533 (36.7)	5945 (33.6)	18,588 (37.8)	
Missing	541	110	431	
Patient gender^c^				
Female	24,870 (49.7)	8865 (49.9)	16,005 (49.6)	0.606
Male	25,149 (50.3)	8908 (50.1)	16,241 (50.4)	
Missing	17,448	24	17,424	
Patient Race/Ethnicity^d^				
White	36,689 (76.4)	8964 (81.3)	27,725 (75.0)	** < 0.01**
Black	6883 (14.3)	1372 (12.4)	5511 (14.9)	
Asian	1003 (2.1)	258 (2.3)	745 (2.0)	
Hispanic	579 (1.2)	0 (0.0)	579 (1.6)	
Other	2865 (6.0)	437 (4.0)	2428 (6.6)	
Missing	19,448	6766	12,682	

*EHR* Electronic Health Record, *SEM* Standard Error of the Mean

^a^Practices were considered adopters if their overall proportion of patient refusals documented was ≥ 5.71%

^b^Characteristics are reported cross-sectionally from values at the end of the Baseline Period. Percentages are calculated among patients with available data; missing data for age group, gender and race/ethnicity was excluded

^c^Information regarding patient gender was only available for 18 clinics; therefore, values are reported among clinics with available patient gender information

^d^Information regarding patient race was only available for 16 clinics; therefore, values are reported among clinics with available patient race information

Boldface indicates *p* < 0.0

*Qualitative Interviews with Champions*. After the Implementation Phase ended, qualitative interviews were conducted with the Physician and Operations Champions and transcribed for the parent study. At least one of the Champions from each practice completed an interview (N = 33). Two questions asked the Champions how useful the assessment and feedback reports were and how they used them to inform their QI projects. The responses that specifically referred to the documentation of patient refusals were selected from the transcripts to review and summarized for the current analysis.

*Study Team Documentation*. The research staff and Sharecare staff reviewed notes from team meetings, coach meetings with practices, and technical assistance requests from practices to summarize the barriers and facilitators to documentation of patient refusals that they observed while implementing the study with participating practices.

*Practice Characteristics*. The number of providers per practice was counted as the number of physicians, advanced practice nurses, and physician assistants. For urbanicity, all practices were located in one of two types of urban areas based on US Census Bureau definitions [35], which represent a densely settled group of Census tracts, differentiated by population size: (1) urbanized area if 50,000 or more (labeled “urban city”), or (2) urbanized cluster if at least 2500 and less than 50,000 (labeled “town/rural area”). Visualize Health data reports included the number of active patients aged 11–17 years by sex, age group, and race/ethnicity.

### Data analysis

For Objective 1, assessing the feasibility of implementing this refusal documentation method, three analyses were performed. The first aimed to identify practice characteristics associated with the practices’ successful adoption of HPV vaccine patient refusal documentation. The mean percentage of eligible well visits with documented patient refusals during the 12-month Implementation Phase across all 21 practices was 5.71%. Based on this mean, practices with greater than or equal to 5.71% of eligible well visits with documented refusals were classified as “adopters,” and practices with less than 5.71% of eligible well visits with documented refusals were considered “non-adopters.” Next, we stratified the practices based on refusal documentation adoption status (adopter vs. non-adopter) and compared the practice characteristics and patient population characteristics between these two categories, using Pearson chi-squared tests for categorial variables and Wilcoxon rank sum tests for continuous variables.

The second analysis under Objective 1 aimed to examine patterns of adoption and maintenance over time by study arm. Among the seven practices that were classified as adopters, we summarized the aggregated proportion of documented refusals during each quarter, overall and for each practice. Pearson chi-squared tests with Yates’ continuity correction were conducted to compare the proportion of documented refusals between the Coach-based and Web-based arms for each quarter. In addition, to confirm whether any observed trends of patient refusals were, in fact, the result of improved documentation and not an artifact of actual decreases in vaccination rates, we calculated Spearman correlations to examine the relationship between monthly rates of vaccination against rates of refusals in each Adopter practice.

The third analysis under Objective 1 was to identify barriers and facilitators of adopting patient refusal documentation. To do this, we summarized the themes identified in the text excerpts from the Champion’s qualitative interviews that mentioned refusal documentation and the themes from the study team documentation and reflections.

Objective 2 was to describe demographic patterns in patient refusals of HPV vaccine. Utilizing the data from the adopter practices, generalized linear mixed models were fit to examine the association between documented patient refusals and patient-level characteristics (age group, gender, and race/ethnicity). Because three adopter practices did not have adequate race/ethnicity information, we conducted two regression models to assess the difference between including and excluding race/ethnicity. Thus, model 1 included age group and gender among all seven adopter practices, while model 2 included age group, gender, and race/ethnicity among the four adopter practices with adequate data.

All statistical analyses were performed in R (R 4.1.1 core team, Vienna, Austria). *p*-values less than 0.05 were considered statistically significant. We used descriptive statistics to describe the practice characteristics as of the end of the Baseline Phase. Because this is secondary analysis of data from a larger study, the sample size was not chosen to achieve certain statistical power. However, with approximately n = 2700 data points from the four practices with full information for model 2, we had excellent power to detect a clinically meaningful odds ratios (OR) for HPV refusal. Specifically, a simple simulation-based power analysis indicated 80% power for OR = 1.5 (or 0.67) for two subgroups of n = 900 if the true proportions are about 15%. Power is 92% if n = 1350 per group for the same parameter setting.

## Results

### Feasibility: correlates of adoption of the patient refusal documentation method

All study practices confirmed that they did not already use a different strategy for documenting refusals of specific vaccines in structured, searchable fields other than the immunization grid prior to the beginning of the practice facilitation implementation, and none expressed interest in exploring a different method during the course of the study. Nine of the 21 practices showed no evidence of any patient refusal documentation during the Baseline or Implementation Phases, and 5 additional practices documented very few refusals, in less than 4% of eligible well visits; thus, these 14 practices were classified as “non-adopters” (Table 1). Only 7 practices met our criteria for classification as an “adopter”. The percent of patients aged 11–12 years was higher in adopter practices than non-adopter practices (adopters = 36.2%, non-adopters = 32.2%, *p* < 0.001). The distribution of patient race/ethnicity differed significantly between the two categories, with a greater percentage of White patients and lower percentage of patients from other racial/ethnic groups in the adopter practices compared to the non-adopter practices (*p* < 0.01). The other practice-level and patient-level characteristics did not significantly differ between adopter and non-adopter practices (*p* > 0.05).

### Feasibility: patterns of adoption and maintenance over time

**Fig. 2:**
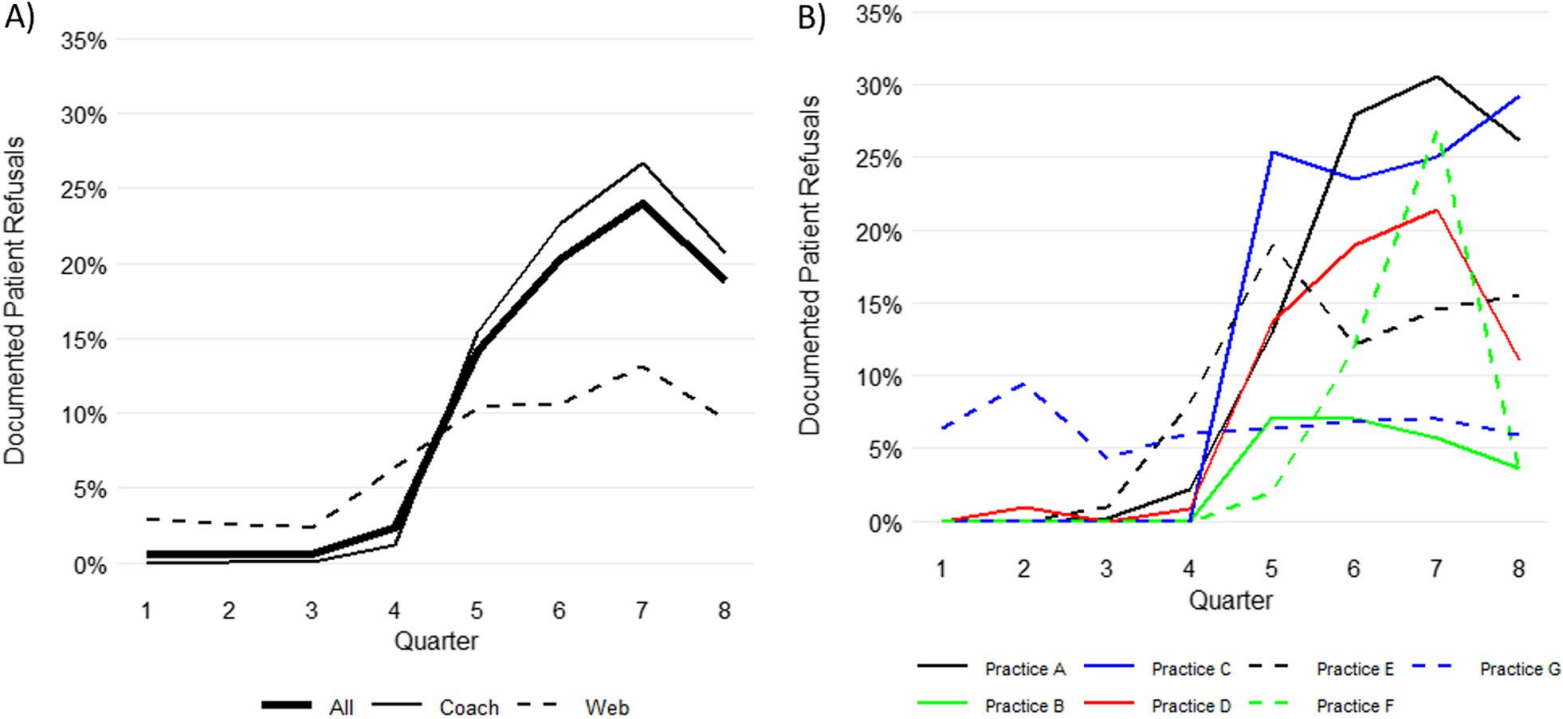
Trends in patient refusal documentation during eligible well visits by (**A**) study arm and (**B**) practice ID during the Baseline Period (Quarters 1–4) and Implementation Phase (Quarters 5–8). *Note*: Thin solid lines are Coach-Based arm and dashed lines are Web-Based arm of the trial

Among the 7 adopter practices, the overall proportion of eligible well visits with documented refusals during the four quarters of the Baseline Phase was less than or equal to 2.4% (Table 2), and the Web-Based arm had higher Baseline percentages than the Coach-Based arm (*p* < 0.001). In contrast, the proportion of eligible well visits with documented refusals increased markedly in the Implementation Phase and was consistently greater than 14% overall across all quarters (Fig. 2A). The highest proportion of documented refusals were during the first QI Project-Quarter 6 (20.2%) and second QI Project-Quarter 7 (24.0%), then the proportion decreased slightly in Quarter 8 (18.8%). The proportions of documented refusals were significantly higher in the Coach-Based practices compared to the Web-Based practices during the Implementation Phase overall (22.4% vs 11.1%) and in each quarter: Quarter 5 (15.4% versus 10.5%), Quarter 6 (22.6% versus 10.6%), Quarter 7 (26.6% vs 13.1%) and Quarter 8 (20.7% vs 9.6%) (all *p* < 0.05).

**Table 2:** Proportion of well visits with documented patient refusals among the seven adopter practices during Baseline Phase and Implementation Phase

Study phase	Proportion of Well Visits with Documented Refusals						*p*-value^a^
	Overall n = 7 Practices		Coach-Based Arm n = 4 Practices		Web-Based Arm n = 3 Practices		
	Refusals / Well Visits	%	Refusals / Well Visits	%	Refusals / Well Visits	%	
*Baseline phase*^b^							
Quarter 1	8/1282	0.62	0/1010	0.00	8/272	2.90	** < 0.001**
Quarter 2	17/2797	0.61	3/2252	0.13	14/545	2.60	** < 0.001**
Quarter 3	14/2278	0.61	2/1764	0.01	12/514	2.30	** < 0.001**
Quarter 4	28/1183	2.40	11/919	1.20	17/264	6.40	** < 0.001**
*Implementation phase*							
QI Project 1^b^							
Quarter 5	182/1283	14.20	150/977	15.40	32/306	10.50	**0.04**
Quarter 6	580/2865	20.20	519/2292	22.60	61/573	10.60	** < 0.001**
QI Project 2^b^							
Quarter 7	623/2591	24.00	558/2094	26.60	65/497	13.10	** < 0.001**
Quarter 8	317/1681	18.80	290/1399	20.70	27/282	9.60	** < 0.001**
Overall for Implementation Phase	1472/8082	18.20	1517/6762	22.40	185/1658	11.10	** < 0.001**

*QI* Quality Improvement

^a^P-value represents the Pearson Chi-square test with Yates’ continuity correction comparing proportion of documented refusals between the coach-based and web-based arms

^b^Each quarter represents 3 months

Boldface indicates *p* < 0.05

As seen in the overall trend for each of the seven adopter practices (Fig. 2B), documentation of refusals increased substantially from the end of Baseline (Quarter 4) to the start of the Implementation Phase (Quarter 5) for all but one practice (Practice G); however, 5 of the 7 practices experienced a decline in patient refusal documentation in Quarter 8. Practice A had the highest proportion of documented refusals overall during the Implementation Phase (27%). Practice C had the second highest refusal documentation during the Implementation Phase (26% overall) and had the highest refusal documentation at the end (Quarter 8; 29%). Practice G showed a unique pattern, starting above the other practices in the Baseline Phase and remaining relatively stable during the Implementation Phase at around 6–7%.

Next, we calculated Spearman correlations between the monthly proportion of eligible well visits with documented refusals and the monthly proportion of eligible well visits with an administered HPV vaccination dose within the 7 adopter practices. We found that 6 out of the 7 adopter practices demonstrated no correlation between HPV vaccine dose administration and refusal documentation (Spearman correlation coefficients ranged from − 0.4 to 0.2), and one was significant (Spearman correlation coefficient = − 0.6 (*p* = 0.05)) (results not shown). Thus, changes in the refusal documentation proportion were most likely due to the inherent improvements in the process of documentation, instead of reflecting changes in patient behavior in HPV vaccine acceptance/refusal.

**Fig. 3:**
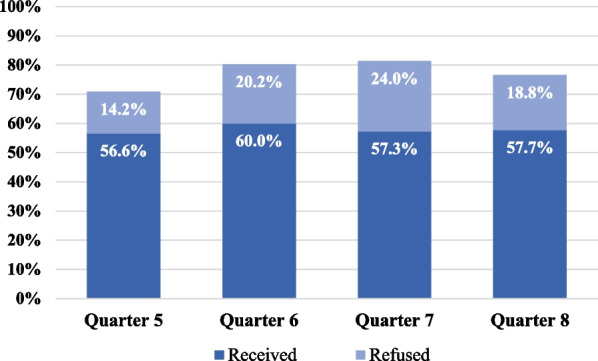
Combination of Visit-Level Data on HPV Vaccine Receipt and Refusal to Measure Provider Recommendation, among Adopter Practices. *Note*: Provider Recommendation = Received + Refused

Figure 3 shows the combination of the two visit-level measures among the seven adopter practices—receipt of an HPV vaccine dose and refusal of HPV vaccine, both calculated as percentages with the same denominator of eligible well visits—to yield the percentage of eligible well visits with documentation of a provider recommendation for HPV Vaccine. Taking into account both doses given and documented refusals, the providers recommended the vaccine in 70.8% to 81.3% percent of eligible well visits.

### Feasibility: qualitative assessment of barriers and facilitators for using the documentation method

Perceptions of the documentation approach utilized for the study contributed to both barriers and facilitators to documenting patient refusals across all study practices (Table 3). Some Champions perceived low utility in making an extra effort to document refusals. However, other Champions indicated that their providers perceived value in the idea of documenting refusals of HPV vaccine so they could see in their data when they were recommending it, even when the patients refused. Some Champions and physicians showed high levels of motivation to document refusals. For example, a physician in Practice A demonstrated interest and created a template within the EHR to record refusals with the dummy codes to be utilized by all practice providers.

**Table 3:** Barriers and facilitators of implementing procedures for documenting patient refusals of HPV vaccination during the practice facilitation of quality improvement projects

Barriers	Facilitators
*Negative perceptions of documentation approach*	*Positive perceptions of documentation approach*
Perceived low utility of extra effort to document patient refusals	Perceived value in having a way to document of patient refusals of specific vaccines
	High motivation to document HPV vaccine refusals
*Workflow integration challenges*	*Workflow integration aids*
Challenging to integrate use of an unfamiliar, non-standard code into existing workflow	Detailed instructions for documenting patient refusals that were provided to practices
Physicians forgetting to use the “dummy code” to document refusals	
Lack of time and low prioritization	Adding manual reminders to document (e.g., laminated card) for physicians within the workflow
Use of “dummy code” complicated the billing process for staff	
*Technical assistance challenges*	*Technical assistance value*
Multiple EHRs across study practices, requiring the software company to customize approaches for integrating documentation of patient refusals into assessment and feedback reports	Practices that requested support for documenting refusals received assistance from the software company in reviewing their process and refusal codes
	Ability to discuss with QI coach whether or not refusals were showing up in assessment and feedback reports

*EHR* Electronic Health Record

Workflow integration challenges created substantial barriers to documentation of patient refusals for many practices. Several champions commented that it was challenging to integrate the use of an unfamiliar, non-standard ICD code into their existing workflow to document refusals outside of the immunization grid or clinical notes. For example, one Operations Champion noted that they had to remind the physicians multiple times to use the dummy codes for refusals, but it was difficult for some of the physicians to change their routines. Some Champions noted that the documentation of refusals was a low priority among providers and they lacked the time to do it. In addition, some practices noted that using the dummy code interfered with the billing process of the practices because some of the claims were rejected due to the way the billing staff had entered the non-billable dummy code. The Physician Champion in Practice F suggested that it would be helpful to receive more instructions for the billing staff on how handle the dummy codes. During meetings with the QI coach, the Practice D Champions explained that their providers had attempted to document refusals with the dummy codes, but the providers became frustrated with the workflow when they ran into difficulties with the billing process did not continue the new documentation workflow when they learned operations staff ran into difficulties with billing.

Workflow integration aids facilitated documentation of patient refusals for some practices. A key facilitator to adopting refusal documentation was the provision of the handout with detailed instructions, particularly when the Champions reviewed these instructions with their providers. Another facilitator was adding manual reminders. For example, one practice created a laminated card listing the dummy codes with a note reminding them to document, then placed the cards on their computers so they would see them during visits.

Technical assistance posed challenges for the software vendor but served as a facilitator for the practices that used it. Because the 21 study practices used 10 different EHRs, customizing the software setup for extracting and integrating their refusal documentation into their assessment and feedback reports was challenging for Sharecare. Several practices took advantage of requesting technical assistance from Sharecare to understand how to document refusals in order for them to show in the assessment and feedback reports. For example, Sharecare assisted Practice F with understanding that the Visualize Health software could not extract refusals being documented in the “miscellaneous/notes tab” of their EHR or in the patient’s clinical notes, so they needed to utilize the dummy codes. In addition, participants in the Coach-Based arm noted the importance of having support of the QI Coach available to the practices to assist in learning how to document refusals and understanding how to interpret these data in their assessment and feedback reports.

### Demographic patterns in patient refusals

In model 1 among the seven adopter practices, adjusting for patient gender, the odds of HPV vaccine refusals for patients aged 15–17 years were 25% higher than the odds for patients aged 11–12 years (Table 4), with no significant difference between patients aged 13–14 and 11–12 years. After adjusting for patient age group, the odds of refusal for male patients were 18% lower than the odds for female patients. In model 2 among the four adopter practices that had data on race/ethnicity, when adjusting for both patient gender and race/ethnicity, the odds of refusals for patients aged 13–14 years and 15–17 years was 39% and 65% higher, respectively, compared to the odds for patients aged 11–12 years. In this model, differences by gender and race/ethnicity were not statistically significant.

**Table 4:** Odds of refusing HPV vaccine during well visits by patient characteristics, among subset of seven practices that adopted refusal documentation

Characteristic	Model 1^a^ Adjusted OR (95% CI)	Model 2^b^ Adjusted OR (95% CI)
Patient age group		
11–12 years	Reference	Reference
13–14 years	1.12 (0.99, 1.28)	**1.39 (1.04, 1.84)**
15–17 years	**1.25 (1.08, 1.43)**	**1.65 (1.23, 2.22)**
Patient gender		
Female	Reference	Reference
Male	**0.82 (0.73, 0.91)**	0.84 (0.66, 1.06)
Patient race		
White	–	Reference
Black	–	0.85 (0.60, 1.19)
Other	–	1.17 (0.47, 2.91)
Unknown	–	0.95 (0.69, 1.33)

*CI* Confidence Interval, *OR* Odds Ratio

^a^Model 1 includes age group and gender only among the 7 adopter practices

^b^Model 2 includes age group, gender, and race; because 3 practices did not have adequate race information, only 4 practices were included in Model 2

Boldface indicates *p* < 0.05

## Discussion

This study assessed the feasibility of a novel method for documenting vaccine refusals using structured fields, with the goal of improving the measurement of provider fidelity to vaccine guidelines while overcoming some of the existing challenges across multiple EHR platforms and billing codes. When we applied the method in our implementation-effectiveness trial focused on practice facilitation and HPV vaccine, we found mixed results for feasibility, with limited adoption but strong use among the adopters, and we demonstrated its ability to characterize patient demographic patterns of HPV vaccine refusals.

Only one-third of the pediatric practices adopted the method, but the adopter practices successfully used it to document refusals in a substantial percentage of well visits. The capture of refusals showed marked increases, up to 24% during the Implementation Phase compared to 2.4% at Baseline. Adopter practices had higher proportions of young patients aged 11–12 years and patients of White race compared to non-adopter practices, but there was no difference in likelihood of adoption based on study arm, practice size, or rural/urban location. Given that the recommended HPV vaccination age begins at 11 years, the practices with higher proportions of adolescents aged 11–12 years may have been more interested in having the ability to document multiple refusals over time, particularly for parents who choose to delay the vaccine until an older age [29, 36]. Practices with higher proportions of patients of white race/ethnicity may have been more interested in refusal documentation due to experiencing more HPV vaccine hesitancy among white adolescents, who have lower rates of HPV vaccination compared to Hispanic and black adolescents in the U.S [15].

One of the facilitators identified for this refusal documentation method was the support of the QI coach. This was consistent with the quantitative data finding that refusal documentation was higher in the Coach-Based versus the Web-Based practice facilitation arm. In addition to coach support, other key facilitators included high levels of motivation, the detailed instructions, and manual reminders. Barriers to refusal documentation included low prioritization, workflow integration, and complication of the billing process. Careful examination of the barriers to adopting vaccine refusal documentation can inform future efforts to improve this method and inform new methods to measure HPV vaccine refusals for QI efforts.

The transition from paper medical charts to EHRs in recent years has been associated with improved documentation and delivery of immunizations [37, 38]. However, EHRs also present barriers to implementation of population vaccination QI efforts due to difficulty in collecting baseline coverage rates or collecting consistent measures across multiple sites with different EHR systems [39]. Even with a common data collection and reporting system supported within this research study provided by Sharecare, site specific adaptations were required. Our experiences were similar to others seeking to improve maternal vaccination rates, which necessitated trying numerous approaches adapted to local conditions to find a solution [24].

These barriers demonstrate a need for EHR systems to adapt more effective documentation of multiple refusals of specific vaccines in structured fields or codes and to enable template reports of visit-level vaccine refusal rates for assessment and feedback. Some providers mentioned that they document vaccine refusals and reasons for refusal in free-text clinic notes for specific visits, but validated natural language processing tools were not available to capture refusals from clinical notes, and the integration of free-text clinical notes with structured data presents many challenges [26, 40]. Despite our novel method to improve vaccine refusal documentation using a combination of the EHR immunization grid and non-billable dummy procedure codes, documentation was difficult to adopt and sustain for some practices. In particular, we found that providers needed additional orientation and reminders of the usefulness of the refusal data to enable tracking their recommendation behaviors, and billing staff needed additional training on dummy code removal from claims before submission to insurance companies. Possible future regulatory requirements on vaccine refusal may necessitate improvement within EHR systems on vaccine refusal documentation.

Among the 7 adopter practices, the odds of refusing the HPV vaccine were higher for older patients (aged 15–17 years) compared to younger (aged 11–12 years), and lower for males compared to females. One possibility for the age pattern could be a higher frequency of providers recommending HPV vaccine at older adolescent visits; i.e., more frequent provider recommendations for older adolescents may be creating more opportunities to refuse [41, 42]. Surveys of physicians have shown that providers recommend HPV vaccination less often to adolescents aged 11–12 compared to older adolescents [31, 43]. Additionally, since HPV vaccine guidelines recommend routine HPV vaccination to adolescents starting at age 11 [44], this older unvaccinated cohort of adolescents may comprise a higher proportion of vaccine-hesitant parents, who may have been offered HPV vaccination earlier and refused.

The finding of lower odds of refusal for males versus females would appear to contradict the pattern of lower HPV vaccine coverage rates for males compared to females seen in the published literature [15, 29]. However, several studies have demonstrated a higher percentage of HPV vaccine acceptance in male adolescents, and they attributed lower male vaccine coverage rates to providers recommending the vaccine less often to males [32, 45]. Additionally, a systematic review identified that males received provider recommendations for HPV vaccines less often than females [46]; thus, it is possible that female adolescents may have higher odds of refusal because they are given more opportunities for being recommended and refusing the vaccine.

There were limitations to our study. The convenience sample of private pediatric practices in Tennessee participating in the parent trial may not be generalizable to other pediatric settings in Tennessee and other states. The qualitative interviews and informal discussions with practice champions, our research team, and Sharecare to identify barriers and facilitators could have been subject to recall bias, halo bias, and recency bias in that interviewees may recall certain events inaccurately or emphasize more recent events over past events.

This study makes important methodological contributions to the fields of informatics and implementation science with implications for future research and practice. We showed that measuring documented refusals in conjunction with documented vaccine administration enables more accurate measurement of provider fidelity to vaccination guidelines. The tenfold increase in refusal documentation showed that a provider recommendation would have been undetected in up to a quarter of visits without counting refusals. Additionally, we demonstrated the feasibility of using this documentation method, successfully using structured EHR fields and codes in seven practices that had 4 different EHRs. At the same time, we identified some challenges in using and sustaining this method due to the dummy codes not being a seamless part of routine documentation during patient encounters and requiring added effort for billing staff. Future studies are needed to further explore additional options for enabling documentation of refusal for HPV vaccine and other specific vaccines using structured EHR fields or codes, with a focus on optimizing workflow integration and sustainability. Providers could give valuable feedback directly to EHR vendors regarding their preferences to enhance refusal documentation as part of the immunization workflow. In addition, while structured data and codes are ideal for generating assessment and feedback reports, future research could assess the feasibility of leveraging natural language processing methods to classify vaccine refusals from free-text clinical notes. Finally, our results suggest that male adolescents are more likely to accept HPV vaccination than females if offered, while older adolescents may require special patient education strategies as they are more likely to be serial refusers.

## Conclusion

A lack of documentation of patient HPV vaccination refusals in structured, searchable fields leads to incomplete information about missed opportunities to vaccinate that could guide QI efforts over time through assessment and feedback reports, and it underestimates clinician’s efforts in appropriately offering vaccinations to an increasing hesitant population. While this specific refusal documentation method using dummy codes had some weaknesses that limited full adoption and sustainability, our findings clearly point to the need for further refinement of refusal measurement methods that could work within the existing structural constraints of our diverse commercial EHR ecosphere and within current patient visit workflows for use in research that focuses on QI-focused implementation strategies. In addition, our findings demonstrate the value of HPV vaccine refusal data and the need for adaptations in EHRs or billing codes. Enabling efficient, streamlined documentation of multiple vaccine refusal instances in structured fields within these systems can create more opportunities for assessment and feedback efforts to improve adherence to HPV vaccination guidelines while informing efforts to address hesitancy.

## Data Availability

The datasets used and/or analyzed during the current study are available from the corresponding author on reasonable request.
